# A Robust Ordering Strategy for Retailers Facing a Free Shipping Option

**DOI:** 10.1371/journal.pone.0125939

**Published:** 2015-05-19

**Authors:** Qing-chun Meng, Xiao-le Wan, Xiao-xia Rong

**Affiliations:** 1 School of Management, Shandong University, Jinan, Shandong, China; 2 School of Mathematics, Shandong University, Jinan, Shandong, China; Nankai University, CHINA

## Abstract

Free shipping with conditions has become one of the most effective marketing tools available. An increasing number of companies, especially e-businesses, prefer to offer free shipping with some predetermined condition, such as a minimum purchase amount by the customer. However, in practice, the demands of buyers are uncertain; they are often affected by many factors, such as the weather and season. We begin by modeling the centralized ordering problem in which the supplier offers a free shipping service and retailers face stochastic demands. As these random data are considered, only partial information such as the known mean, support, and deviation is needed. The model is then analyzed via a robust optimization method, and the two types of equivalent sets of uncertainty constraints that are obtained provide good mathematical properties with consideration of the robustness of solutions. Subsequently, a numerical example is used to compare the results achieved from a robust optimization method and the linear decision rules. Additionally, the robustness of the optimal solution is discussed, as it is affected by the minimum quantity parameters. The increasing cost-threshold relationship is divided into three periods. In addition, the case study shows that the proposed method achieves better stability as well as computational complexity.

## Introduction

With the rapid development of e-commerce and the logistics industry, the free shipping strategy implemented by e-business enterprises has become an effective approach to attract and retain customers. Currently, most e-commerce companies, especially business-to-consumer (B2C) and business-to-business (B2B) enterprises, offer free shipping to buyers who spend more than a specified amount of money. An increasing number of electric commercial enterprises have begun adopting this free shipping strategy. For example, Amazon, the globally recognized online bookstore, offers free shipping to customers when a purchase meets or exceeds 35 dollars. E-business enterprises in China, Tianmao and Jingdong, also apply a free shipping strategy if a certain purchase amount is met. The growth and evolution of the e-commerce sector have undeniably highlighted the importance of shipping and handling (S&H) fees to business models. Suppliers can gain cost advantages in order processing and implementation by lowering the frequency of small shipments. Furthermore, if many retailers order jointly, the free shipping requirement may result in cost savings. Therefore, free shipping strategies have become a subject of interest and important research topic for both suppliers and consumers. As the survey evidence indicates, more than 50% of online shoppers complain about shipping fees, and more than 60% of shoppers will cancel an order if shipping fees increase. Research has shown that fulfillment issues are a crucial driver of customer satisfaction.

In this paper, the order centralization problem with free shipping appears to be important for attracting customers and increasing turnover. Given that a supplier and multiple retailers whose demands for commodities have an uncertain coexistence in a supply chain, a retailer may primarily focus on how free shipping schedules influence ordering strategies. Lewis et al. [1] used an ordered probability model to confirm the effects of nonlinear and discontinuous free shipping on purchasing decisions. The model shows that retailers are quite sensitive to shipping charges. In addition, promotion policies, such as free shipping and free shipping for orders that exceed a certain pre-determined quantity or amount threshold, are quite effective in generating additional sales. Some researchers considered the pricing and free shipping strategies of B2C and B2B companies by modeling two-stage decision problems [2, 3]. These studies performed sensitivity analyses to test this influence and discussed the leader-follower action based on game theory. Gumus et al. [4] studied the partitioning decisions of retailers using a two-scenario analysis and illustrated that the prices charged by retailers are relevant to their shipping fee polices. Scholars have also investigated the management of stochastic inventory systems with a free shipping option [5]. Abad and Aggarwal [6] studied a pricing decision with random demand to reduce transport costs that includes free shipping with conditions. Hua et al. [7] addressed the optimal order strategy of a retailer whose demand is either deterministic or stochastic when suppliers provide a free shipping service. These researchers analyzed the effects of transportation costs on the retailer’s optimal order strategy based on the EOQ model and the newsvendor model. Academics and practitioners presented the centralization ordering problem of retailers with stochastic demands that require limited distributional information, such as the known mean and support as well as several deviation measures of the random data, and suppliers offer free shipping only when the total order amount reaches a certain threshold [8].

One issue in the above study is the same assumption of distributional information about random data. Because this information is difficult to obtain in practice, this limitation has stimulated interest in robust optimization (RO) as an alternative approach to handling uncertain data. In RO, compared with full distributional knowledge, which is difficult to obtain, only partial information such as the known mean, support, and deviation is needed. In contrast to the analysis in [8], which considers the basic linear rule method, uncertain demand via the RO method is considered in this paper.

The RO method was first proposed by Soyster in [9], which used math set theory to describe the uncertain information of the parameters. This method uses the large probability of avoiding decision deviation in poor conditions to obtain the robust domain and avoid large losses. This method can effectively avoid the instability of other algorithms and has great potential application value. For example, Ben-Tal [10] applied the RO method to the dynamic management of supply contracts. Bertsimas and Thiele [11] also applied the RO method to the inventory-pricing model and inventory management. Bertsimas et al. [12] surveyed the theoretical and applied study of RO and highlighted applications of RO across various fields and subjects, such as finance, machine learning, and many areas of engineering. Therefore, adopting the RO method to analyze the optimal ordering model with free shipping under uncertain demand is feasible. We thus propose a new optimization algorithm to solve this problem.

The remainder of the paper is structured as follows. A stochastic programming model of the optimal order strategy of retailers will be presented in *Section Problem Descriptions*, with uncertain demands and a free shipping option. In *Section Robust Optimization*, based on the linear decision rule and the affine assumption, the robust counterpart of the stochastic programming model is analyzed, and a new equivalent determined model is formulated. In *Section Numerical Experiments*, a numerical experiment confirms that the result based on robust analysis is better than that obtained in [8]. In addition, the level of sensitivity is analyzed in terms of how order incidence is affected by the size threshold of free shipping, and the difference in individual and joint chance constraints is also considered. Finally, the conclusion in the final section summarizes this study and provides several recommendations.

## Problem Descriptions

According to the study by Meng et al. [8], we consider the same problem in which a supplier and a number of retailers trade certain types of goods. The supplier offers the goods to retailers whose demands for commodities are uncertain. All retailers order goods uniformly, and the order price is constant. Only when the total order amount reaches a certain threshold can the supplier offer free shipping to retailers. In this problem, we consider how to minimize the costs to all retailers by selecting their optimal order quantity. Here, all retailers are rational, and their inventories are inadequate to meet real demand. In addition, no competition exists among retailers, who are willing to participate in the buying group to pay the total minimum fee. Therefore, the reasonable objective of this problem is to minimize the total cost of all retailers subject to condition that the demands of all retailers are met.

To model the problem in stochastic programming, we denote the following notions, which are the same as those in [8]. The notions m and c are the retail price and order price of the goods, respectively, and q is the known threshold for free shipping. The random demand of the *i*th retailer is $d(\tilde{z})$, which is independent from the others, and *l*_*i*_ is the current inventory, where *i* = 1, 2, ⋯, *n* is the number of retailers. We denote the order quantity *x*_*i*_ as the decision variable. Only when $\sum_{i=1}^{n}x_{i}\geq q$ is met can the supplier offer free shipping to retailers; otherwise, the retailers should assume a cost $f\left(\sum_{i=1}^{n}x_{i}\right)$. The symbol $w_{i}(\tilde{z})$ is a decision variable representing the shortage of goods caused by $d_{i}(\tilde{z})$ in an uncertain environment. The symbol *y* is a 0–1 variable in which 1 means payoff for the transport and 0 is free shipping. Therefore, the model of the optimal order strategy with a free shipping option is given as follows (1).

$$\begin{array}{l} \text{min}\;c\times (\sum_{i=1}^{n}x_{i})+f(\sum_{i=1}^{n}x_{i})\times y+m\times E(\sum_{i=1}^{n}w(\tilde{z}))\\ s.t.\left\{ \begin{array}{l} x_{i}+w_{i}(\tilde{z})\geq d_{i}(\tilde{z})-l_{i},i=1,2,\cdots n\\ x_{i},w_{i}(\tilde{z})\geq 0,i=1,2,\cdots n\\ y=0,if\sum_{i=1}^{n}x_{i}\geq q\\ y=1,if\sum_{i=1}^{n}x_{i}<q \end{array}\right. \end{array}$$ (1)

Where the objective function contains the ordering cost, transportation cost, and penalty cost generated if the demands are not satisfied. For the penalty cost to be relevant to the uncertain realization of $\tilde{z}$, a certain expectation applies here. According to the objective function, the order quantity of the goods must not be so high that it adds inventory, and the shortage must not be sufficiently high to increase costs. The first constraint means that for the *i*th retailer, the sum of the ordering quantity, shortage, and inventory quantity should be not less than their demand. The second constraint shows that the decision variables of ordering quantity and shortage are nonnegative. The third constraint ensures that the ordering quantity is not less than the given threshold *q*; otherwise, the retailers should pay for the transportation cost.

In this paper, we assume that the random demand $d(\tilde{z})$ and the random shortage $w_{i}(\tilde{z})$ are general uncertain variables whose distributional functions or other full knowledge are unknown and for which limited distributional information is known. Perhaps the most appropriate way to ensure a constraint is to restrict its upper bound of violation probability. Such a constraint is regarded as a probabilistic or chance constraint. According to the idea of chance constraint in stochastic programming, the first constraint that $x_{i}+w_{i}(\tilde{z})+l_{i}\geq d_{i}(\tilde{z})$ should be feasible with a probability of at least 1 – *β* and can be rewritten as $P(x_{i}+w_{i}(\tilde{z})+l_{i}\geq d_{i}(\tilde{z}))\geq 1-\beta$, where *β* is a small probability.

One critical issue of chance-constrained problems involves determining the distributional condition relevant to the stochastic data. An individual chance-constrained condition second-order-cone representable only for certain special distributions. That is, the original optimization model is equivalent to a second-order-cone model, which is computationally intractable. However, for general distributions, chance-constrained problems are difficult to solve computationally and are ranked as NP-hard problems [13]. However, the RO method has perfectly illustrated the approximate computation of chance-constrained problems. Therefore, we use the RO method to analyze model (1).

## Robust Optimization

### 3.1. Related knowledge

RO is a more recent and distinct approach in uncertainty optimization. It mainly focuses on traditional optimization theory and tractability, with modeling power and structural results excluded. Besides, the RO has achieved wide practice in different disciplines and fields, such as finance, investment, machine learning, logistics and engineering. According to [14], it is feasible to analyze the solvability of the problem via the linear decision rule. Therefore, we assume that the recourse variable $w_{i}(\tilde{z})$ is the linear function relying on a set of independent random variables *z*_*k*_, *k* = 1,⋯,*N*, as

$$w(\tilde{z})=w^{0}+\sum_{k=1}^{N}w^{k}{\tilde{z}}_{k},{\tilde{z}}_{k}\in W,W=(-{\underline{z}}_{i},{\bar{z}}_{i})$$ (2)

Where *w* ^0^ is the nominal value of $w(\tilde{z})$, *w* ^*k*^ is the direction of data perturbation, and ${\tilde{z}}_{k}$ is the primitive uncertainty with mean zero and support in $\left(-{\underline{z}}_{i},{\bar{z}}_{i}\right)$. Meanwhile, according to the general robust analysis, parameter data $d(\tilde{z})$ are affinely dependent on ${\tilde{z}}_{k}$ as $d(\tilde{z})=d^{0}+\sum_{k=1}^{N}d^{k}\tilde{z}$. Similarly, *d* ^0^ is the nominal value of $d(\tilde{z})$, and *d* ^*k*^ is the direction of data perturbation. Then, $x_{i}+w_{i}(\tilde{z})\geq d_{i}(\tilde{z})-l_{i}$ can be rewritten as

$$x_{i}+w_{i}^{0}+\sum_{k=1}^{N}w_{i}^{k}{\tilde{z}}_{k}\geq d_{i}^{0}+\sum_{k=1}^{N}d_{i}^{k}{\tilde{z}}_{k}-l_{i}$$ (3)

In this paper, because *u* ∈ *R* ^*n*^ is a vector, we denote the definition of the vector norm and dual norm according to [15, 16]. The vector norm is adopted in the form of an uncertainty set: ‖*u*‖ = ‖|*u*|‖ and ‖*u*‖ ≤ ‖*u*‖_2_ for ∀*u*, where |*u*| is the vector with the j component equal to |*u*_*j*_| for each j from 1 to *n*. We refer to this norm as an absolute norm. The dual norm ‖⋅‖^*^ is defined as ${\left\|u\right\|}^{*}=\underset{\left\|x\right\|\leq 1}{\text{max}}u'x$.

We then describe several basic properties from the literature [15, 16] that are used to prove the conclusion of theorems 4 and 5.

#### Proposition 1

For the absolute norm,

$$\begin{array}{l} \left(\text{a}\right)\;one\;has\;{\left\|u\right\|}^{*}={\left\||u|\right\|}^{*};\\ \left(\text{b}\right)\;for\;all\;v,u,\;such\;that\;\left|v\right|\leq \left|u\right|,{\left\|v\right\|}^{*}\leq {\left\|u\right\|}^{*};\\ \left(\text{c}\right)\;for\;all\;v,u,\;such\;that\;\left|v\right|\leq \left|u\right|,\|v\|\leq \|u\|;\\ \left(\text{d}\right)\;{\left\|u\right\|}^{*}\geq {\left\|u\right\|}_{2},\forall u. \end{array}$$ (4)

#### Proposition 2

*For the programming as follows*,

$$\begin{array}{l} z^{*}=\text{max}\;a'v+b'u\\ s.t.\left\{ \begin{array}{l} \left\|v+u\right\|\leq \delta \\ v,u\geq 0 \end{array}\right. \end{array}$$ (5)

*For a*, *v*, *b*, *u* ∈ *R* ^*n*^, *the optimal value is z* ^*^ = *δ*‖*t*‖^*^, *where t*_*i*_ *=* max{*a*_*i*_, *b*_*i*_, 0}, *i* = 1⋯*n*.

In optimization models, if random variables are difficult to corporate, then operation becomes difficult and often computationally intractable. The purpose is to obtain probability bounds for constraint violations rather than to analyze complete distributional information. Here, we denote the set of values associated with the forward and backward deviations of a random variable $\tilde{z}$. Let $\tilde{z}$ be a random variable, and let $M_{\tilde{z}}(s)=E(\text{exp}(s\tilde{z}))$ be its moment-generating function. We denote the set of forward deviations of $\tilde{z}$ as follows:

$$F(\tilde{z})=\{\alpha :\alpha \geq 0,M_{\tilde{z}-E(\tilde{z})}(\frac{\phi }{\alpha })\leq \text{exp}(\frac{\phi^{2}}{\alpha }),\forall \phi \geq 0.$$ (6)

Similarly, the following set is defined for backward deviations.

$$B(\tilde{z})=\{\alpha :\alpha \geq 0,M_{\tilde{z}-E(\tilde{z})}(-\frac{\phi }{\alpha })\leq \text{exp}(\frac{\phi^{2}}{2}),\forall \phi \geq 0.$$ (7)

When $\tilde{z}$ is symmetrically distributed around its mean, we have $F(\tilde{z})=B(\tilde{z})$.

#### Proposition 3

*Let* $\tilde{x}$ *and* $\tilde{y}$ *be two independent random variables with zero means, such that* $p_{\tilde{x}}\in F(\tilde{x})$, $q_{\tilde{x}}\in B(\tilde{x})$, $p_{\tilde{y}}\in F(\tilde{y})$ *and* $q_{\tilde{y}}\in B(\tilde{y})$.

- *If* $\tilde{z}=\tilde{x}+\tilde{y}$, *then* $(p_{\tilde{z}},q_{\tilde{z}})=(\sqrt{p_{\tilde{x}}^{2}+p_{\tilde{y}}^{2}},\sqrt{q_{\tilde{x}}^{2}+q_{\tilde{y}}^{2}}$ *satisfies* $p_{\tilde{z}}\in F(\tilde{z})$, $q_{\tilde{z}}\in B(\tilde{z})$.
- $p(\tilde{x}>\Omega p_{\tilde{x}})\leq \text{exp}(-\Omega^{2}/2)$ *and* $p(\tilde{x}<-\Omega q_{\tilde{x}})\leq \text{exp}(-\Omega^{2}/2)$.

We will analyze the primitive uncertainty $\tilde{z}$ in two cases. The first case addresses only the norm uncertainty set, which is computed relatively easily. In the second case, we will discuss the constraint of *W* in (2) at the base of the norm uncertainty set, which has greater accuracy and complexity than that in the first case.

### 3.2. Robust analysis based on the norm uncertainty set

If $\tilde{z}$ is symmetrically distributed, then we use the following symmetrical norm set, $A_{1}=\{\tilde{z}:\left\|\tilde{z}\right\|\leq \Omega \}$, where Ω ∈ *R* is a fixed number. Generally, if $\tilde{z}$ is asymmetrically distributed, then we may use the asymmetrical norm uncertainty set as follows: $A_{2}=\{\tilde{z}:\tilde{z}=\tilde{v}-\tilde{u},\left\|P^{-1}\tilde{v}+Q^{-1}\tilde{u}\right\|\leq \Omega ,\tilde{v},\tilde{u}\geq 0,\tilde{v},\tilde{u}\in R^{N}\}$, where *P* = *diag*(*p*_1_⋯*p*_*N*_), *Q* = *diag*(*q*_1_⋯*q*_*N*_), *p*_*i*_, *q*_*i*_ > 0. Specifically, when *P*, *Q* are identity matrices, the set *A*_2_ is equivalent to *A*_1_. Then, the constraint (3) is equivalent to

$$x_{i}+w_{i}^{0}+\sum_{k=1}^{N}w_{i}^{k}({\tilde{v}}_{k}-{\tilde{u}}_{k})\geq d_{i}^{0}+\sum_{k=1}^{N}d_{i}^{k}({\tilde{v}}_{k}-{\tilde{u}}_{k})-l_{i},\left\|P^{-1}\tilde{v}+Q^{-1}\tilde{u}\right\|\leq \Omega ,\tilde{v},\tilde{u}\geq 0$$ (8)

#### Theorem 4

For $\Omega =\sqrt{2\;\text{ln}(1/\beta )}$, ∃*P* = *diag*(*p*_1_⋯*p*_*N*_), *Q* = *diag*(*q*_1_⋯*q*_*N*_), *t* ∈ *R* ^*N*^ if *x* satisfies its robust counterpart as follows:

$$\left\{ \begin{array}{l} -x_{i}-w_{i}^{0}+\Omega {\left\|t\right\|}^{*}\leq -d_{i}^{0}+l_{i}\\ t_{i}^{k}\geq p_{k}(d_{i}^{k}-w_{i}^{k})\\ t_{i}^{k}\geq -q_{k}(d_{i}^{k}-w_{i}^{k}) \end{array}\right.$$ (9)

then $P\{x_{i}+w_{i}(\tilde{z})\geq d_{i}(\tilde{z})-l_{i}\}\geq 1-\beta$ holds.

Proof. We first express how to obtain the set (9).

According to (8), for the *i*th retailer, we have

$$-x_{i}-w_{i}^{0}+\sum_{k=1}^{N}\left(d_{i}^{k}-w_{i}^{k}\right)({\tilde{v}}_{k}-{\tilde{u}}_{k})\leq -d_{i}^{0}+l_{i}\;\text{and}\;\left\|P^{-1}\tilde{v}+Q^{-1}\tilde{u}\right\|\leq \Omega ,\tilde{v},\tilde{u}\geq 0$$ (10)

Formula (10) holds if and only if

$$-x_{i}-w_{i}^{0}+\underset{\left\{\tilde{v},\tilde{u}:\left\|P^{-1}\tilde{v}+Q^{-1}\tilde{u}\right\|\leq \Omega ,\tilde{v},\tilde{u}\geq 0\right\}}{\text{max}}\{\sum_{k=1}^{N}\left(d_{i}^{k}-w_{i}^{k}\right)({\tilde{v}}_{k}-{\tilde{u}}_{k})\}\leq -d_{i}^{0}+l_{i}$$ (11)

According to Proposition 2, we have

$$\begin{array}{c} -x_{i}-w_{i}^{0}+\Omega {\left\|t\right\|}^{*}\leq -d_{i}^{0}+l_{i},\\ \text{where}\;t=(t_{1},t_{2}\cdots ,t_{N})\in R^{N},\;t_{k}=\text{max}\{d_{i}^{k}-w_{i}^{k})p_{k},-(d_{i}^{k}-w_{i}^{k})q_{k},0\}. \end{array}$$ (12)

Therefore, a new set known as the robust counterpart is obtained.

$$\left\{ \begin{array}{l} -x_{i}-w_{i}^{0}+\Omega {\left\|t\right\|}^{*}\leq -d_{i}^{0}+l_{i}\\ t_{k}\geq p_{k}(d_{i}^{k}-w_{i}^{k})\\ t_{k}\geq -q_{k}(d_{i}^{k}-w_{i}^{k}) \end{array}\right.$$

Next, we prove that *x* satisfies (9) if and only if $P(x_{i}+w_{i}(\tilde{z})\geq d_{i}(\tilde{z})-l_{i})\geq 1-\beta$ holds.

$$\begin{array}{l} P\{x_{i}+w_{i}(\tilde{z})\leq d_{i}(\tilde{z})-l_{i}\}\\ =P\{x_{i}+w_{i}^{0}+\sum_{k=1}^{N}w_{i}^{k}{\tilde{z}}^{k}\leq d_{i}^{0}+\sum_{k=1}^{N}d_{i}^{k}{\tilde{z}}^{k}-l_{i}\}\\ =P\{\sum_{k=1}^{N}(d_{i}^{k}-w_{i}^{k}){\tilde{z}}^{k}\geq -d_{i}^{0}+l_{i}+x_{i}+w_{i}^{0}\} \end{array}$$

From (12) and *Proposition* 1(d), we have $-d_{i}^{0}+l_{i}+x_{i}+w_{i}^{0}\geq \Omega {\left\|t\right\|}^{*}\geq \Omega {\left\|t\right\|}_{2}$, such that the following inequality related to the probability above holds.

$$P\{x_{i}+w_{i}(\tilde{z})\leq d_{i}(\tilde{z})-l_{i}\}\leq P\{\sum_{k=1}^{N}(d_{i}^{k}-w_{i}^{k}){\tilde{z}}^{k}\geq \Omega {\left\|t\right\|}^{*}\}\leq P\{\sum_{k=1}^{N}(d_{i}^{k}-w_{i}^{k}){\tilde{z}}^{k}>\Omega {\left\|t\right\|}_{2}\}$$ (13)

With the elements of *P*, *Q* from the sets $P(\tilde{z})$, $Q(\tilde{z})$, according to *Proposition 3* (b), ${\left\|t\right\|}_{2}\in P\left(\sum_{k=1}^{N}(d_{i}^{k}-w_{i}^{k}){\tilde{z}}^{k}\right)$ holds. Additionally, when $\Omega =\sqrt{2\;\text{ln}(1-\beta )}$ holds, we have

$$P\{x_{i}+w_{i}(\tilde{z})\leq d_{i}(\tilde{z})-l_{i}\}\leq \text{exp}(-\Omega^{2}/2)=\beta$$

Therefore, $P\{x_{i}+w_{i}(\tilde{z})\geq d_{i}(\tilde{z})-l_{i}\}\geq 1-\beta$ holds.

In this case, *A*_1_, *A*_2_ is discussed only when considering the absolute norm Ω. The distribution of the random variable is not computed, including the constraint of ${\tilde{z}}_{i}\in W$ and $W=(-\underline{z},\bar{z})$ in (3); thus, there is some deviation between the counterpart set and the original set. Furthermore, we consider the distribution *W* of the random variable based on the norm uncertainty set.

### 3.3 The robust analysis based on the norm uncertainty set and *W*

In this case, we consider all probable values of $d(\tilde{z}),W(\tilde{z}),$ including the worst-case $W=(-\underline{z},\bar{z})$. The constraint of $\tilde{z}$ in the norm uncertainty set *A*_1_ may be written as

$$B_{1}=\left\{\tilde{\xi }:\left\|\xi \right\|\leq \Omega ,-\underline{z}\leq \xi \leq \bar{z}\right\}$$

Corresponding to *A*_2_, we have

$$B_{2}=\left\{\xi :\xi =\tilde{v}-\tilde{u},\left\|P^{-1}\tilde{v}+Q^{-1}\tilde{u}\right\|\leq \Omega ,-\underline{z}\leq \tilde{v}-\tilde{u}\leq \bar{z},\tilde{v},\tilde{u}\geq 0\right\}$$

Where *P* = *diag*(*p*_1_,⋯,*p*_*N*_) and *Q* = *diag*(*q*_1_,⋯,*q*_*N*_) with *p*_*i*_, *q*_*i*_ > 0,*i* = 1,⋯,*N*, such that the constraint (3) can be expressed as follows

$$\begin{array}{c} x_{i}+w_{i}^{0}+\sum_{k=1}^{N}w_{i}^{k}({\tilde{v}}_{k}-{\tilde{u}}_{k})\geq d_{i}^{0}+\sum_{k=1}^{N}d_{i}^{k}({\tilde{v}}_{k}-{\tilde{u}}_{k})-l_{i},\;\text{with}\\ \left\|P^{-1}\tilde{v}+Q^{-1}\tilde{u}\right\|\leq \Omega ;\tilde{v}=({\tilde{v}}_{i}^{k},\cdots ,{\tilde{v}}_{i}^{k})\in R_{+}^{N},{\tilde{u}}_{i}^{k}=({\tilde{u}}_{i}^{k},\cdots {\tilde{u}}_{i}^{k})\in R_{+}^{N};-\underline{z}\leq {\tilde{v}}_{i}^{k}-{\tilde{u}}_{i}^{k}\leq \bar{z} \end{array}$$ (14)

Similarly, we reach the following conclusions.

#### Theorem 5

For *P* = *diag*(*p*_1_,⋯,*p*_*N*_), *Q* = *diag*(*q*_1_,⋯ *q*_*N*_), *t* ∈ *R* ^*N*^, *u* ∈ *R* ^*l*^, $\Omega =\sqrt{2\;\text{ln}(1/\beta )}$ if *x* satisfies the robust counterpart as follows

$$\left\{ \begin{array}{l} -x_{i}-w_{i}^{0}+\underset{r,s\geq 0}{\text{min}}\left\{\Omega {\left\|t^{i}\right\|}^{*}+r_{i}\bar{z}+s_{i}\underline{z}\right\}\leq d_{i}^{0}+l_{i}\\ t_{k}^{i}\geq p_{k}(d_{i}^{k}-w_{i}^{k}-r_{i}^{k}+s_{i}^{k}),k=1,2,\cdots ,N\\ t_{k}^{i}\geq -q_{k}(d_{i}^{k}-w_{i}^{k}-r_{i}^{k}+s_{i}^{k}),k=1,2,\cdots ,N\\ r_{i}^{k},s_{i}^{k}\geq 0,k=1,2,\cdots ,N,i=1,2,\cdots ,n \end{array}\right.$$ (15)

then $P\{x_{i}+w_{i}(\tilde{z})\geq d_{i}(\tilde{z})-l_{i}\}\geq 1-\beta$ holds, where *β* is a small number.

Proof. We first explain how to obtain the set (15). Let

$$\varphi_{i}=(\varphi_{i}^{1},\varphi_{i}^{2},\cdots ,\varphi_{i}^{N})=(d_{i}^{1}-w_{i}^{1},d_{i}^{2}-w_{i}^{2},\cdots ,d_{i}^{N}-w_{i}^{N}),$$

Thus, (14) can be rewritten as $-x_{i}-w_{i}^{0}+\sum_{k=1}^{N}\left(d_{i}^{k}-w_{i}^{k}\right)({\tilde{v}}_{i}^{k}-{\tilde{u}}_{i}^{k})\leq -d_{i}^{0}+l_{i}$, with $\left\|P^{-1}\tilde{v}+Q^{-1}\tilde{u}\right\|\leq \Omega ,\tilde{v},\tilde{u}\geq 0,-\underline{z}\leq {\tilde{v}}_{i}^{k}-{\tilde{u}}_{i}^{k}\leq \bar{z}$, which holds if and only if the following inequality holds.

$$-x_{i}-w_{i}^{0}+\underset{\left\{\tilde{v},\tilde{u}:\left\|P^{-1}\tilde{v}+Q^{-1}\tilde{u}\right\|\leq \Omega ,-\underline{z}\leq {\tilde{v}}_{i}^{k}-{\tilde{u}}_{i}^{k}\leq \bar{z},\tilde{v},\tilde{u}\geq 0\right\}}{\text{max}}\left\{\sum_{k=1}^{N}\varphi_{i}^{k}({\tilde{v}}_{i}^{k}-{\tilde{u}}_{i}^{k})\right\}\leq -d_{i}^{0}+l_{i}$$

According to strong duality theory, we can obtain the equivalent representation

$$\begin{array}{l} \Leftrightarrow -x_{i}-w_{i}^{0}+\underset{r,s\geq 0}{\text{min}}\left\{\underset{\left\{\tilde{v},\tilde{u}:\left\|P^{-1}\tilde{v}+Q^{-1}\tilde{u}\right\|\leq \Omega ,\tilde{v},\tilde{u}\geq 0\right\}}{\text{max}}\left\{\sum_{k=1}^{N}\left\{\varphi_{i}^{k}({\tilde{v}}_{i}^{k}-{\tilde{u}}_{i}^{k})+r_{i}^{k}(\bar{z}-{\tilde{v}}_{i}^{k}+{\tilde{u}}_{i}^{k})+s_{k}(\underline{z}+{\tilde{v}}_{i}^{k}-{\tilde{u}}_{i}^{k})\right\}\right\}\right\}\leq -d_{i}^{0}+l_{i}\\ \Leftrightarrow -x_{i}-w_{i}^{0}+\underset{r,s\geq 0}{\text{min}}\left\{\underset{\left\{\tilde{v},\tilde{u}:\left\|P^{-1}\tilde{v}+Q^{-1}\tilde{u}\right\|\leq \Omega ,\tilde{v},\tilde{u}\geq 0\right\}}{\text{max}}\left\{(\varphi_{i}-r_{i}+s_{i})•{\tilde{v}}_{i}-(\varphi_{i}-r_{i}+s_{i})•{\tilde{u}}_{i}+\bar{z}(\sum_{k=1}^{N}r_{i}^{k})+\underline{z}(\sum_{k=1}^{N}s_{i}^{k})\right\}\right\}\leq -d_{i}^{0}+l_{i} \end{array}$$ (16)

In the last inequality, *r*, *s* are irrelevant to $\tilde{v},\tilde{u}$, such that we have

$$\begin{array}{l} \underset{\left\{\tilde{v},\tilde{u}:\left\|P^{-1}\tilde{v}+Q^{-1}\tilde{u}\right\|\leq \Omega ,\tilde{v},\tilde{u}\geq 0\right\}}{\text{max}}\left\{(\varphi_{i}-r_{i}+s_{i})•{\tilde{v}}_{i}-(\varphi_{i}-r_{i}+s_{i})•{\tilde{u}}_{i}+\bar{z}(\sum_{k=1}^{N}r_{i}^{k})+\underline{z}(\sum_{k=1}^{N}s_{i}^{k})\right\}\\ =\underset{\left\{\tilde{v},\tilde{u}:\left\|P^{-1}\tilde{v}+Q^{-1}\tilde{u}\right\|\leq \Omega ,\tilde{v},\tilde{u}\geq 0\right\}}{\text{max}}\left\{(\varphi_{i}-r_{i}+s_{i})•{\tilde{v}}_{i}-(\varphi_{i}-r_{i}+s_{i})•{\tilde{u}}_{i}\right\}+\bar{z}(\sum_{k=1}^{N}r_{i}^{k})+\underline{z}(\sum_{k=1}^{N}s_{i}^{k}) \end{array}$$ (17)

When the formula above is inserted into (16), according to Proposition 2, (16) has the equivalent representation

$$-x_{i}-w_{i}^{0}+\underset{r,s\geq 0}{\text{min}}\left\{\Omega {\left\|t^{i}\right\|}^{*}+\bar{z}(\sum_{k=1}^{N}r_{i}^{k})+\underline{z}(\sum_{k=1}^{N}s_{i}^{k})\right\}\leq -d_{i}^{0}+l_{i}$$ (18)

Where $t_{k}(r,s)=\text{max}\left\{(\varphi_{i}^{k}-r_{i}^{k}+s_{i}^{k})p_{k}-(\varphi_{i}^{k}-r_{i}^{k}+s_{i}^{k})q_{k},0\right\}$

Therefore, a new set known as the robust counterpart with the form (15) is obtained.

$$\left\{ \begin{array}{l} -x_{i}-w_{i}^{0}+\underset{r,s\geq 0}{\text{min}}\left\{\Omega {\left\|t^{i}\right\|}^{*}+\bar{z}(\sum_{k=1}^{N}r_{i}^{k})+\underline{z}(\sum_{k=1}^{N}s_{i}^{k})\right\}\leq -d_{i}^{0}+l_{i}\\ t_{k}^{i}\geq p_{k}(d_{i}^{k}-w_{i}^{k}-r_{i}^{k}+s_{i}^{k})\\ t_{k}^{i}\geq -q_{k}(d_{i}^{k}-w_{i}^{k}-r_{i}^{k}+s_{i}^{k})\\ r_{i}^{k},s_{i}^{k}\geq 0,k=1,2,\cdots ,N,i=1,2,\cdots ,n \end{array}\right.$$

Next, we will prove that $\Omega =\sqrt{2\;\text{ln}(1/\beta )}$ if and only if $P\{x_{i}+w_{i}(\tilde{z})\geq d_{i}(\tilde{z})-l_{i}\}\geq 1-\beta$.

From (18), we have

$$\begin{array}{l} P\{x_{i}+w_{i}(\tilde{z})\geq d_{i}(\tilde{z})-l_{i}\}\\ =P\left(\sum_{k=1}^{N}(d_{i}^{k}-w_{i}^{k}){\tilde{z}}^{k}\geq -d_{i}^{0}+l_{i}+x+w_{i}^{0}\right)\\ \leq P\left(\sum_{k=1}^{N}(d_{i}^{k}-w_{i}^{k}){\tilde{z}}^{k}\geq \underset{r,s\geq 0}{\text{min}}\left\{\Omega {\left\|t^{i}\right\|}^{*}+\bar{z}(\sum_{k=1}^{N}r_{i}^{k})+\underline{z}(\sum_{k=1}^{N}s_{i}^{k})\right\}\right)\\ \leq P(\sum_{k=1}^{N}(d_{i}^{k}-w_{i}^{k}){\tilde{z}}^{k}\geq \underset{r,s\geq 0}{\text{min}}\left\{\Omega {\left\|t\right\|}_{2}+\bar{z}(\sum_{k=1}^{N}r_{i}^{k})+\underline{z}(\sum_{k=1}^{N}s_{i}^{k})\right\} \end{array}$$ (19)

Where the last inequality follows from Proposition 1. Let *t* ^*^ = *t*(*r* ^*^, *s* ^*^) be the optimal solution of $\underset{r,s\geq 0}{\text{min}}\left\{\Omega {\left\|t\right\|}_{2}+\bar{z}(\sum_{k=1}^{N}r_{i}^{k})+\underline{z}(\sum_{k=1}^{N}s_{i}^{k})\right\}$, and considering the inequality $\xi \in (-\underline{z},\bar{z})$, we have

$$\begin{array}{l} P\left(\sum_{k=1}^{N}(d_{i}^{k}-w_{i}^{k}){\tilde{z}}^{k}\geq \underset{r,s\geq 0}{\text{min}}\left\{\Omega {\left\|t^{i}\right\|}_{2}+\bar{z}(\sum_{k=1}^{N}r_{k})+\underline{z}(\sum_{k=1}^{N}s_{k})\right\}\right)\\ =P\left(\sum_{k=1}^{N}(d_{i}^{k}-w_{i}^{k}){\tilde{z}}^{k}\geq \Omega {\left\|t^{i^{*}}\right\|}_{2}+\bar{z}(\sum_{k=1}^{N}r_{i}^{k^{*}})+\underline{z}(\sum_{k=1}^{N}s_{i}^{k^{*}})\right)\\ =P(\sum_{k=1}^{N}(\varphi_{i}^{k}-r_{i}^{k^{*}}+s_{i}^{k^{*}})){\tilde{z}}^{k}\geq \Omega {\left\|t^{i^{*}}\right\|}_{2} \end{array}$$ (20)

With the elements of *P*, *Q* from sets $P(\tilde{z})$, $Q(\tilde{z})$, according to Proposition 3, we have ${\left\|t_{i}^{*}\right\|}_{2}\in P\left((\varphi_{i}^{k}-r_{i}^{k^{*}}+s_{i}^{k^{*}})\xi \right)$. Therefore, for $\Omega =\sqrt{2\;\text{ln}(1/\beta )}$, the following inequality holds:

$$P\{x_{i}+w_{i}(\tilde{z})\leq d_{i}(\tilde{z})-l_{i}\}\leq \text{exp}(-\Omega^{2}/2)=\beta$$

Therefore, $P\{x_{i}+w_{i}(\tilde{z})\leq d_{i}(\tilde{z})-l_{i}\}\geq 1-\beta$ holds.

Thus, we obtain two types of robust counterparts for problem (1) with uncertain demand, where the chance constraint is transformed into a convex group of linear or second-order-cone constraints. Therefore, the transformed program will yield a global optimum solution to the problem.

## Numerical Experiments

Assume that three retailers order goods from the same supplier. The retail price of a unit of a good is m = 70, and the order price is c = 40. The transportation function is proportional to the order quantity, that is, r = 4. The given threshold is *q* = 60. The current inventories of the retailers are 5, 8, and 10, respectively. Their demands are in the same form $d(\tilde{z})=30+\tilde{z}$, that is, $d_{i}^{0}=30$, $d_{i}^{1}=1$. The recourse variable $w(\tilde{z})$ is in the form $w(\tilde{z})=w^{0}+w^{1}\tilde{z}$, where *w* ^0^ is the usual value of $w(\tilde{z})$ and *w* ^1^ is the direction of data perturbation. Here, $\tilde{z}$ is the primitive uncertainty for which the known mean is zero and the support is $\tilde{z}\in [-1,2]$.

According to section 3.2, the robust program of this detailed problem is

$$\begin{array}{l} \text{min}\;40\times (\sum_{i=1}^{3}x_{i})+4\times (\sum_{i=1}^{3}x_{i})\times y+70\times (\sum_{i=1}^{3}w_{i}^{0})\\ s.t.\left\{ \begin{array}{l} -x_{1}-w_{1}^{0}+\Omega {\left\|t^{1}\right\|}^{*}\leq -30+5\\ -x_{2}-w_{2}^{0}+\Omega {\left\|t^{2}\right\|}^{*}\leq -30+8\\ -x_{3}-w_{3}^{0}+\Omega {\left\|t^{3}\right\|}^{*}\leq -30+10\\ t_{1}^{1}=\text{max}\{p_{1}(1-w_{1}^{1}),-q_{1}(1-w_{1}^{1}),0\}\\ t_{1}^{2}=\text{max}\{p_{1}(1-w_{2}^{1}),-q_{1}(1-w_{2}^{1}),0\}\\ t_{1}^{3}=\text{max}\{p_{1}(1-w_{3}^{1}),-q_{1}(1-w_{3}^{1}),0\}\\ x_{i}\geq 0,i=1,2,3\\ y=0,if\sum_{i=1}^{3}x_{i}\geq 60\\ y=1,if\sum_{i=1}^{3}x_{i}<60 \end{array}\right. \end{array}$$ (21)

According to section 3.3, the robust program of this detailed problem is as follows

$$\begin{array}{l} \text{min}\;40\times (\sum_{i=1}^{3}x_{i})+4\times (\sum_{i=1}^{3}x_{i})\times y+70\times (\sum_{i=1}^{3}w_{i}^{0})\\ s.t.\left\{ \begin{array}{l} -x_{1}-w_{1}^{0}+\underset{r,s\geq 0}{\text{min}}\{\Omega {\left\|t^{1}\right\|}^{*}+r_{1}^{1}-2s_{1}^{1}\}\leq -30+5\\ -x_{2}-w_{2}^{0}+\underset{r,s\geq 0}{\text{min}}\{\Omega {\left\|t^{2}\right\|}^{*}+r_{2}^{1}-2s_{2}^{1}\}\leq -30+8\\ -x_{3}-w_{3}^{0}+\underset{r,s\geq 0}{\text{min}}\{\Omega {\left\|t^{3}\right\|}^{*}+r_{3}^{1}-2s_{3}^{1}\}\leq -30+10\\ t_{1}^{1}\geq \text{max}\{p_{1}(1-w_{1}^{1}-r_{1}^{1}+s_{1}^{1}),-q_{1}(1-w_{1}^{1}-r_{1}^{1}+s_{1}^{1}),0\}\\ t_{1}^{2}=\text{max}\{p_{1}(1-w_{2}^{1}-r_{2}^{1}+s_{2}^{1}),-q_{1}(1-w_{2}^{1}-r_{2}^{1}+s_{2}^{1}),0\}\\ t_{1}^{3}=\text{max}\{p_{1}(1-w_{3}^{1}-r_{3}^{1}+s_{3}^{1}),-q_{1}(1-w_{3}^{1}-r_{3}^{1}+s_{3}^{1}),0\}\\ x_{i},r_{i}^{1},s_{i}^{1}\geq 0,i=1,2,3\\ y=0,if\sum_{i=1}^{3}x_{i}\geq 60\\ y=1,if\sum_{i=1}^{3}x_{i}<60 \end{array}\right. \end{array}$$ (22)

First, we compute the forward and backward deviations. Based on the approximate method in [15], we obtain *p*_1_ = 0.96 and *q*_1_ = 1.5.

We then consider all *β*_*i*_ = 0.001 (that is, the stochastic demand of every retailer can be guaranteed with the probability 1 − *β* = 99.999%), and $\Omega =\sqrt{2\;\text{ln}(1/\beta )}=3.72$ is obtained. Solving model (21) with the above data using LINGO solver, we find that the optimal ordering strategies for the three retailers are 22, 21.34, and 20, respectively, and the optimal cost value is 2700, in which the free shipping condition is satisfied for the total order amount of 63.34. By solving model (22) with the above data, we find that the optimal ordering strategies for the three retailers are 22.93, 20.42, and 16.97, respectively, and the optimal cost value is 2413, in which the free shipping condition is satisfied for the total order amount of 60.3. The solution for model (22) is obviously superior to that for model (21); this finding illustrates that the analysis in section 3.3 based on the norm uncertainty set and *W* is more accurate.

Regarding this same numerical experiment, the solution is far superior to that used in [8], in which the optimal value was 2770. Therefore, compared with the linear decision method, the robust analysis in this paper is more scientific because the counter-constraint by the linear decision method is linear in [8]. However, the robust counterparts of this paper’s model are conic quadratic constraints with a higher degree of complexity.

Furthermore, for the more accurate model (22), we consider the sensitivity of the effect of threshold *q* on the optimal value. Initially, we use the value five times the current value (i.e., 60), and we present the optimal function values in Table 1. This result shows that the optimal cost value is constant from *q* = 40 to *q* = 50, increases from *q* = 50 to *q* = 70, and then becomes constant again from *q* = 70 to *q* = 80.

**Table 1:** The Relationship between the Threshold and the Optimal Cost.

Threshold of *q*	40	45	50	55	60	65	70	75	80
Optimal cost	2056	2056	2056	2217	2413	2724	2986	2986	2986

**Fig 1:**
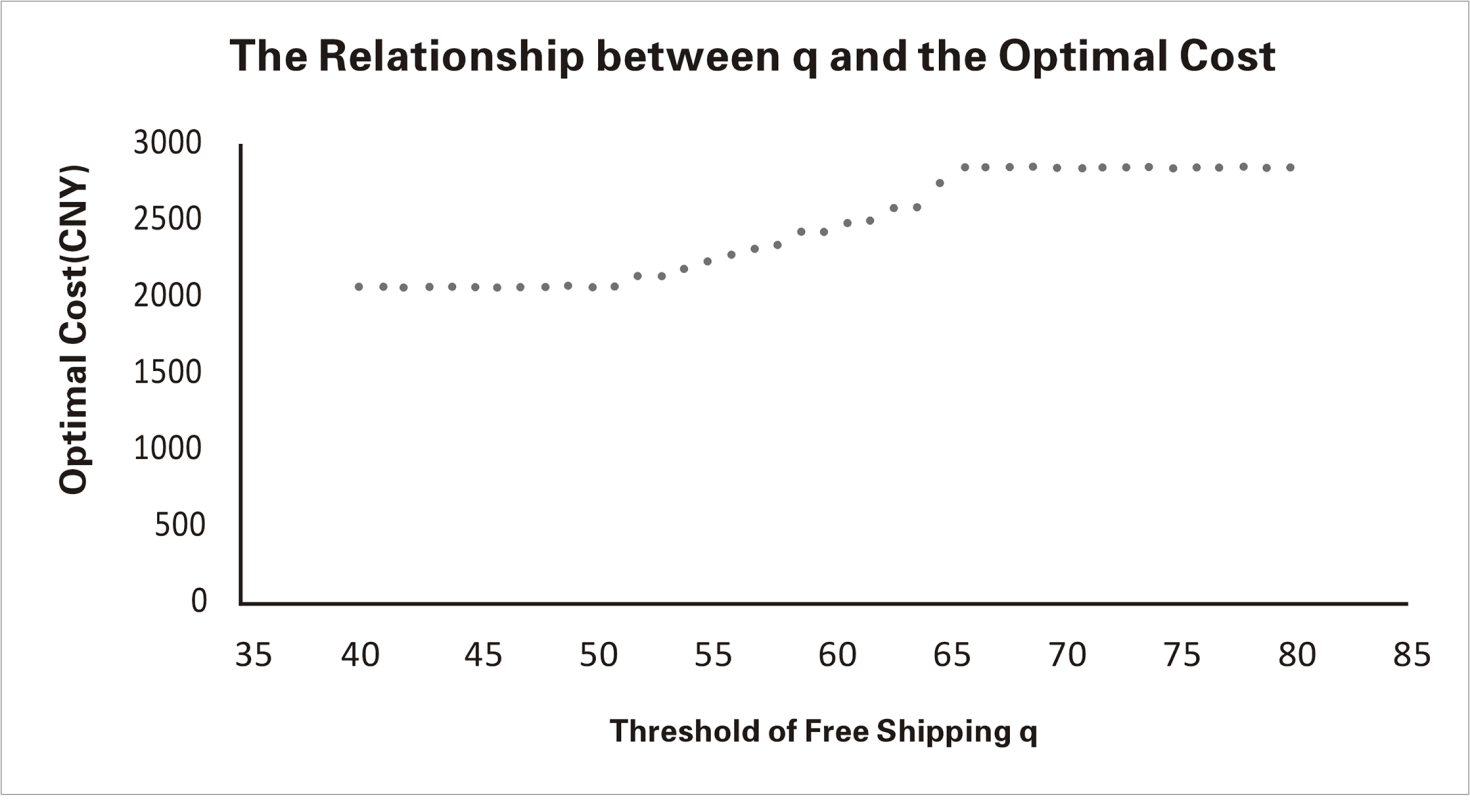
The Detailed Relationship between the Threshold and the Optimal Value.

To find the relationship, we consider 40 groups of thresholds and optimal values (*q* = 40, 41,…, 80) in greater detail, as shown in Fig 1.

**Fig 2:**
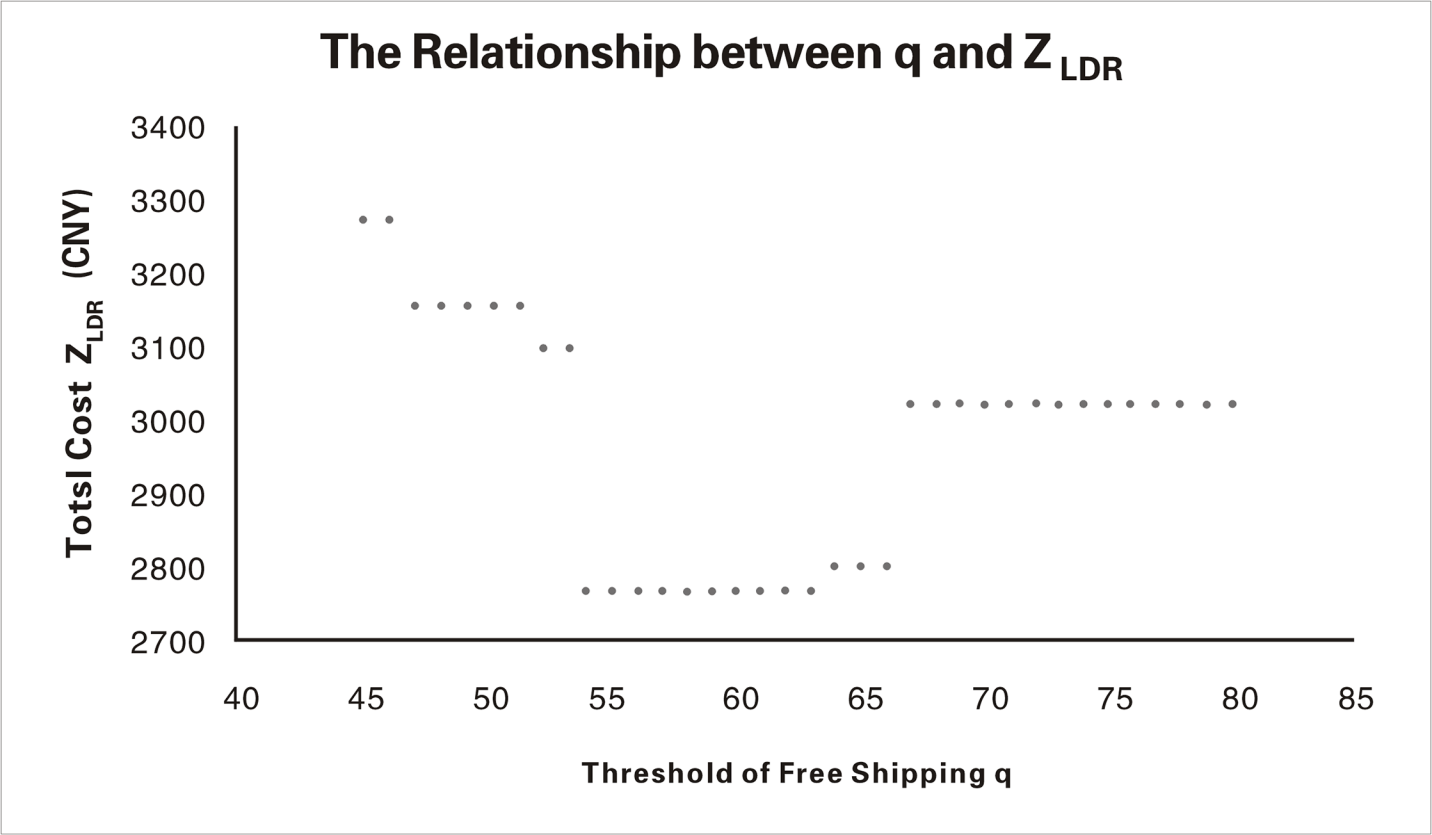
The Detailed Relationship between the Threshold and the Optimal Value in [8].

A comparison between Fig 1 and Fig 2 reveals that the optimal cost obtained by the RO method in this paper is much more accurate. Additionally, this value varies less when the size threshold changes, indicating better sensitivity.

Finally, in both (21) and (22), the stochastic demand of every retailer can be guaranteed with the probability 1 – *β* = 99.999%, which is known as individual chance constraints. If each retailer coordinates strategies with other retailers, a new question arises in that the demand of all retailers is guaranteed with a probability that is not less than 1 – *β*, that is, $P\{x_{i}+w_{i}(\tilde{z})\leq d_{i}(\tilde{z})-l_{i},i=1,2,3\}\leq \beta$. This constraint is defined as a joint chance constraint, which is different from $P\{x_{i}+w_{i}(\tilde{z})\leq d_{i}(\tilde{z})-l_{i}\}\leq \beta_{i},i=1,2,3$. In many studies, the typical approach involves choosing *β*_*k*_ = *β*/*n* subject to $P\{x_{i}+w_{i}(\tilde{z})\leq d_{i}(\tilde{z})-l_{i}\}\leq \beta_{i}$, $\sum_{i=1}^{3}\beta_{i}\leq \beta$ [17]. Here, using *β* = 0.001 and n = 3, we obtain $\Omega =\sqrt{2\;\text{ln}(1/3\beta_{i})}=3.4$. The optimal costs in models (21) and (22) are 2700 and 2477, respectively, which shows no obvious differences from the result found for individual chance constraints.

## Conclusions

Understanding the effect of free shipping services on a business model is a critical component of supply chain management (SCM). This study provides a swift, cost-effective and efficient guide for scientific decision making and support in e-commerce activities. This paper studied the optimal order problem under demand uncertainty and proposed a stochastic programming model in which the objective function is to minimize the total cost of all retailers. Because of the limited information regarding the uncertain variables in the model, this study adopted the RO method to analyze the chance constraint and finds that the two types of equivalence sets used for the uncertainty constraint obtained are tractable. Finally, this paper compares the results obtained from the RO method with those obtained based on the linear decision rule. The findings indicate that the RO method is more effective in terms of computational complexity and stability.
